# Supplementary material for: Intestine-specific removal of DAF-2 nearly doubles lifespan in Caenorhabditis elegans with little fitness cost
Source: Nat Commun. 2022 Oct 25;13:6339. doi: 10.1038/s41467-022-33850-4 (PMC9596710; doi:10.1038/s41467-022-33850-4)
Supplement: Supplementary file 1 — Supplementary Information [file 41467_2022_33850_MOESM1_ESM.pdf]

Supplementary information

Intestine-specific removal of DAF-2 nearly doubles lifespan in *Caenorhabditis elegans* with little fitness cost

Authors: Yan-Ping Zhang[1, 2#], Wen-Hong Zhang[1, 2#], Pan Zhang[1], Qi Li[3], Yue Sun[1], Jia-Wen Wang[1], Shaobing O. Zhang[3], Tao Cai[1], Cheng Zhan[1], Meng-Qiu Dong[1, 2*]

Affiliations:

1. National Institute of Biological Sciences, Beijing, Beijing, China
2. Beijing Key Laboratory of the Cell Biology of Animal Aging, Beijing, China
3. Laboratory of Metabolic Genetics, College of Life Sciences, Capital Normal University, Beijing, China

[#] *These authors contributed equally to this work.*

* *Correspondence:* dongmengqiu@nibs.ac.cn.

Supplementary Fig. 1. *daf-16::gfp* and *daf-2::mNeonGreen* knock-in alleles have no effect on lifespan (a) or development (b). Related to Fig. 1 and 2.

Supplementary Fig. 2. Expression of DAF-2::mNeonGreen and *daf-2*::NuGFP. Related to Fig. 1.

Supplementary Fig. 3. The spatiotemporal expression pattern of DAF-16::GFP*.* Related to Fig. 2.

Supplementary Fig. 4. Strain construction for tissue-specific AID by CRISPR/Cas9 genome editing. Related to Fig. 3 and 4.

Supplementary Fig. 5. Tissue-specific AID of DAF-2::degron::mNeonGreen. Related to Fig. 3.

Supplementary Fig. 6. Tissue-specific AID of DAF-16::GFP::degron in *daf-2(e1370)* worms. Related to Fig. 4.

Supplementary Fig. 7. WT lifespan is not affected by 1 mM auxin treatment. Related to Fig. 3 and 4.

Supplementary Fig. 8. Degradation of neuronal DAF-2 by AID is sustained in old worms. Related to Fig. 3.

Supplementary Fig. 9. Survival curves of *daf-2(e1370)* and DAF-2 AID worms fed on either live or dead bacteria. Related to Fig. 3.

Supplementary Fig. 10. Transient dauers induced by degrading intestinal DAF-2 at 27 °C. Related to Fig. 5a.

Supplementary Fig. 11. Transcriptome analysis of tissue-specific DAF-2 AID worms. Related to Fig. 6.

Supplementary Fig. 12. Enrichment of KEGG terms in the transcriptomic changes induced by tissue-specific DAF-2 AID, using GSEA. Related to Fig. 6.

Supplementary Fig.13. Tissue-specific transcriptome analysis of the intestinal DAF-2 AID worms. Related to Fig. 7.

Supplementary Fig. 14. Examination of the tissue specificity of two neuron-specific promoters *rgef-1* and *rab-3*, using transgenic NuGFP reporters. Related to Fig. 3.

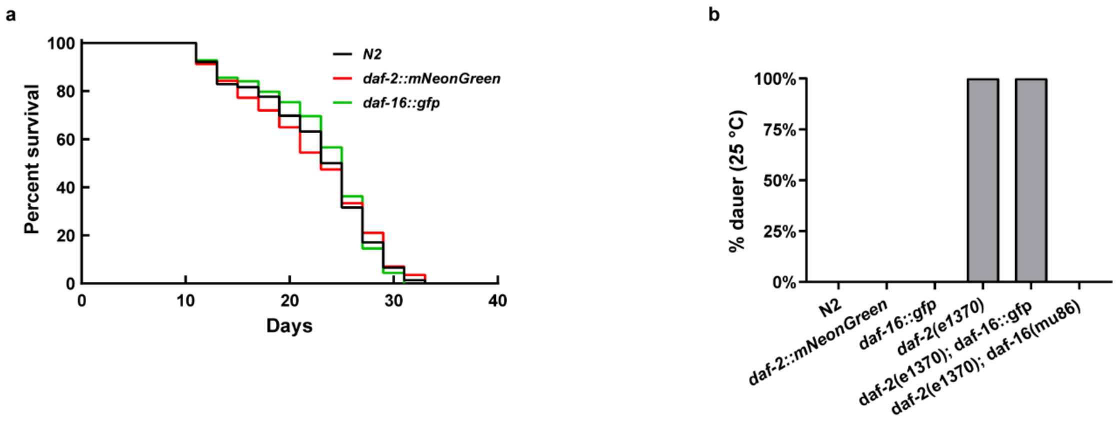

Supplementary Fig. 1. *daf-16::gfp* and *daf-2::mNeonGreen* knock-in alleles have no effect on lifespan (a) or development (b). Related to Fig. 1 and 2.

See survival statistics in Supplementary Data 1 (**a**) and data points used for the derivation of data (**b**) in Source Data.

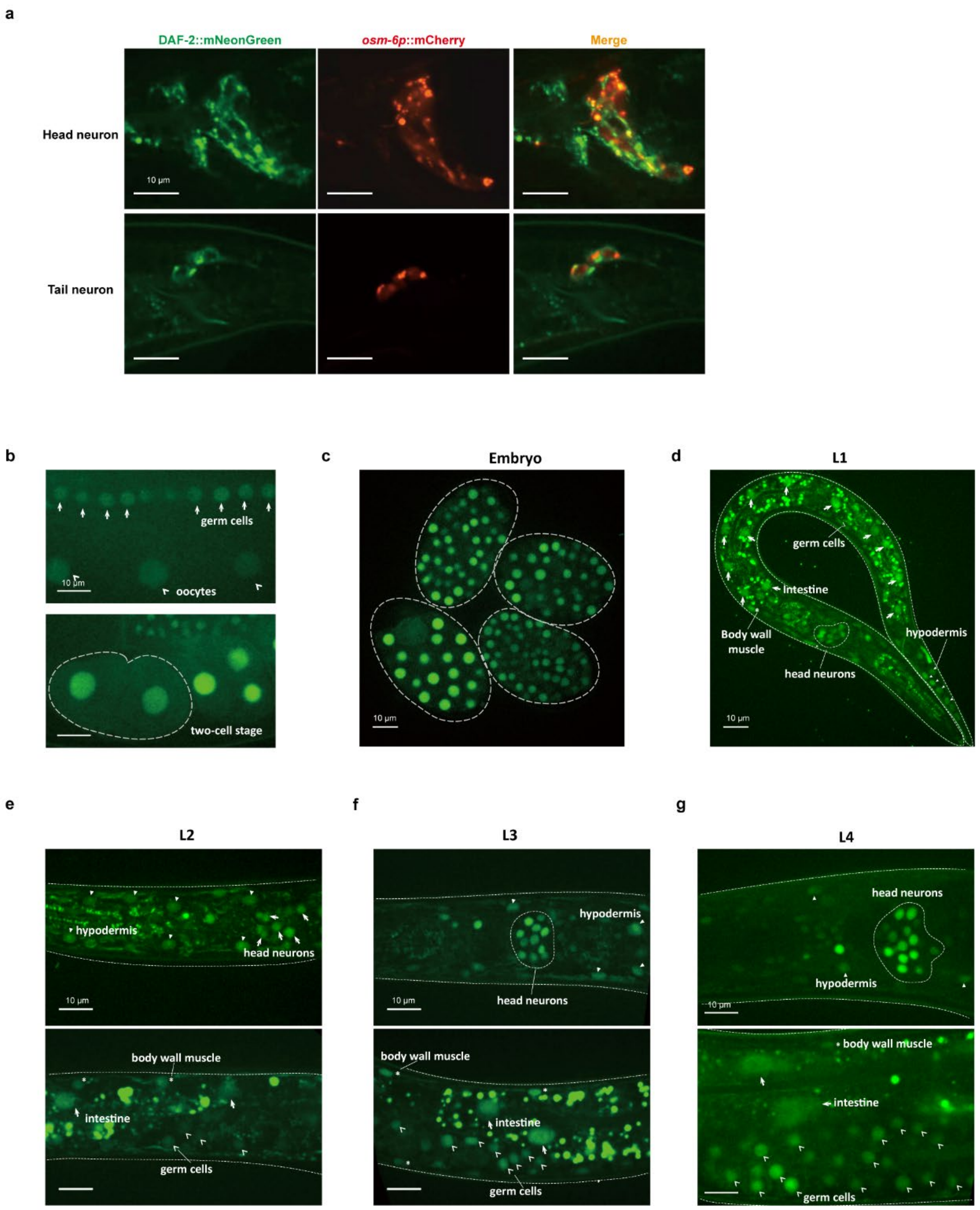

Supplementary Fig. 2. Expression of DAF-2::mNeonGreen and *daf-2*::NuGFP. Related to Fig. 1.

a Sensory neurons marked by *osm-6p*::mCherry express DAF-2::mNeonGreen. A similar pattern of expression was observed in two independent experiments. **b-g** The spatiotemporal expression pattern of *daf-2*::NuGFP*.* A similar pattern of expression was observed in two independent experiments.

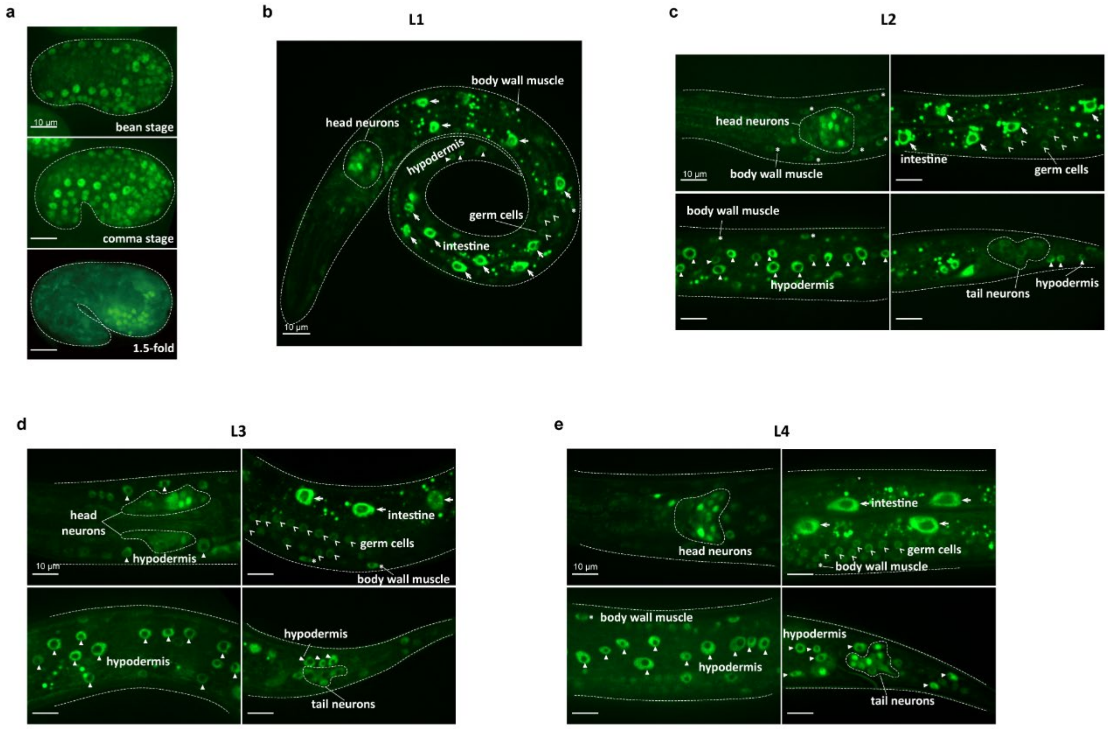

Supplementary Fig. 3. The spatiotemporal expression pattern of DAF-16::GFP. Related to Fig. 2.

a-e The expression pattern of DAF-16::GFP at embryonic stages (**a**), L1 larval stage (**b**), L2 larval stage (**c**), L3 larval stage (**d**), and L4 larval stage (**e**). A similar pattern of expression was observed in three independent experiments.

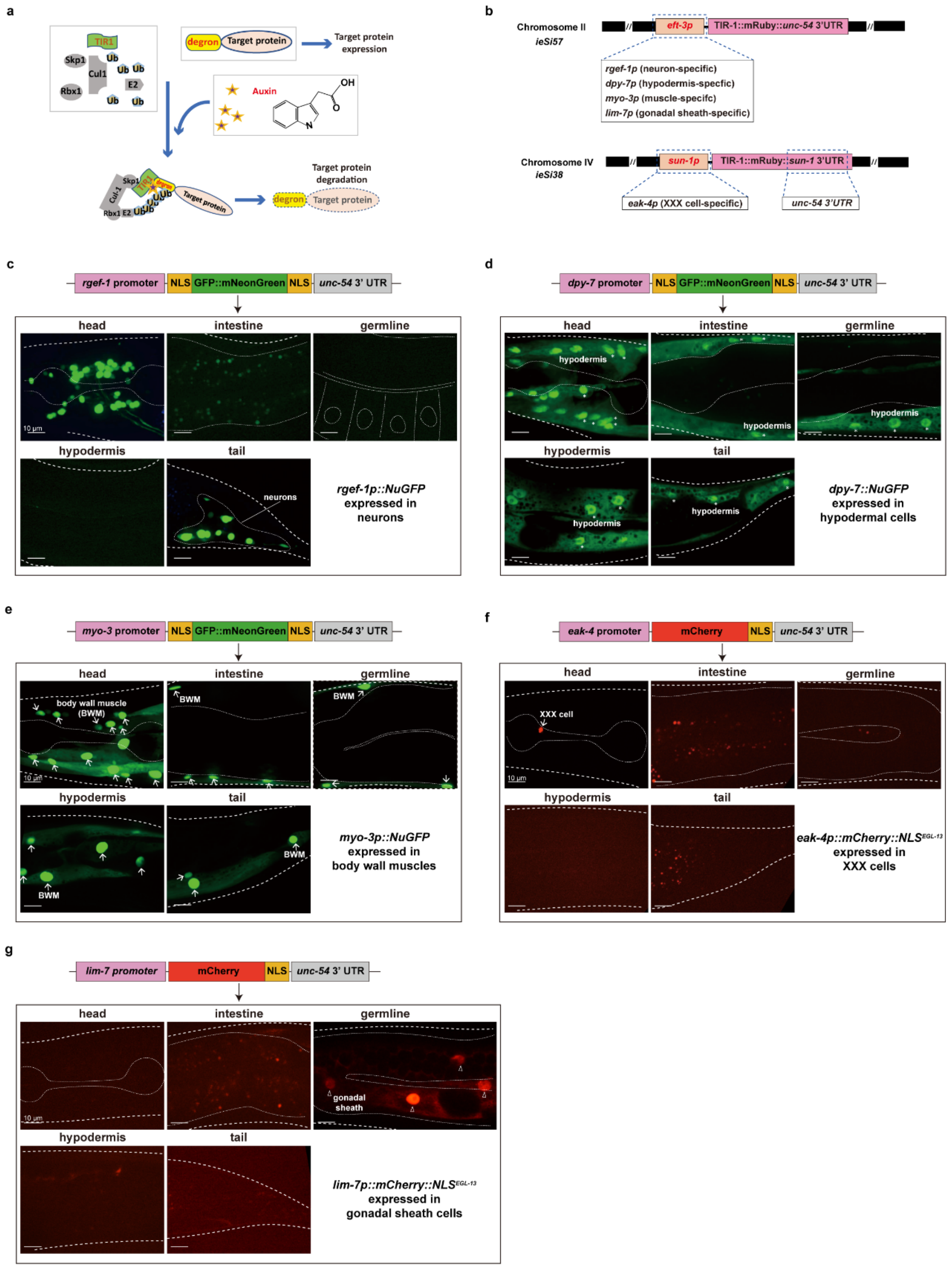

Supplementary Fig. 4. Strain construction for tissue-specific AID by CRISPR/Cas9 genome editing. Related to Fig. 3 and 4.

a Targeted protein degradation using the auxin-inducible degron system. **b** Construction of five additional tissue-specific TIR-1-expressing strains. **c-g** Verification of the tissue specificity of the five promoters using NuGFP or mCherry as a reporter. A similar pattern of expression was observed in two independent experiments.

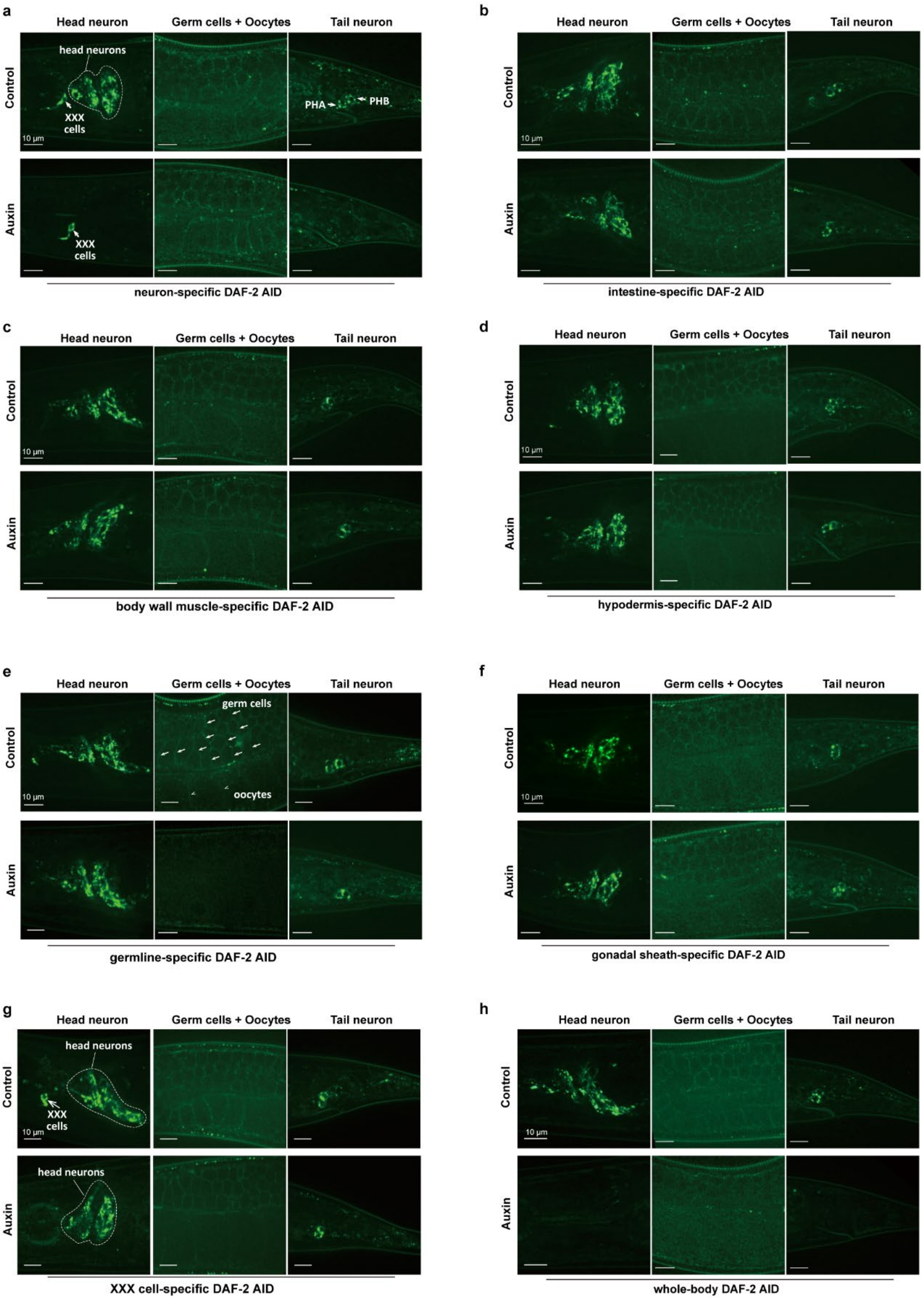

Supplementary Fig. 5. Tissue-specific AID of DAF-2::degron::mNeonGreen. Related to Fig. 3.

a-h Specifically degrading DAF-2 in the neurons (**a**), intestine (**b**), body wall muscles (**c**), hypodermis (**d**), germline (**e**), gonadal sheath (**f**), XXX cells (**g**), or whole body (**h**)

with 1 mM auxin treatment for 24 hours starting from late L4 stage at 20 °C. A similar pattern of expression was observed in two independent experiments.

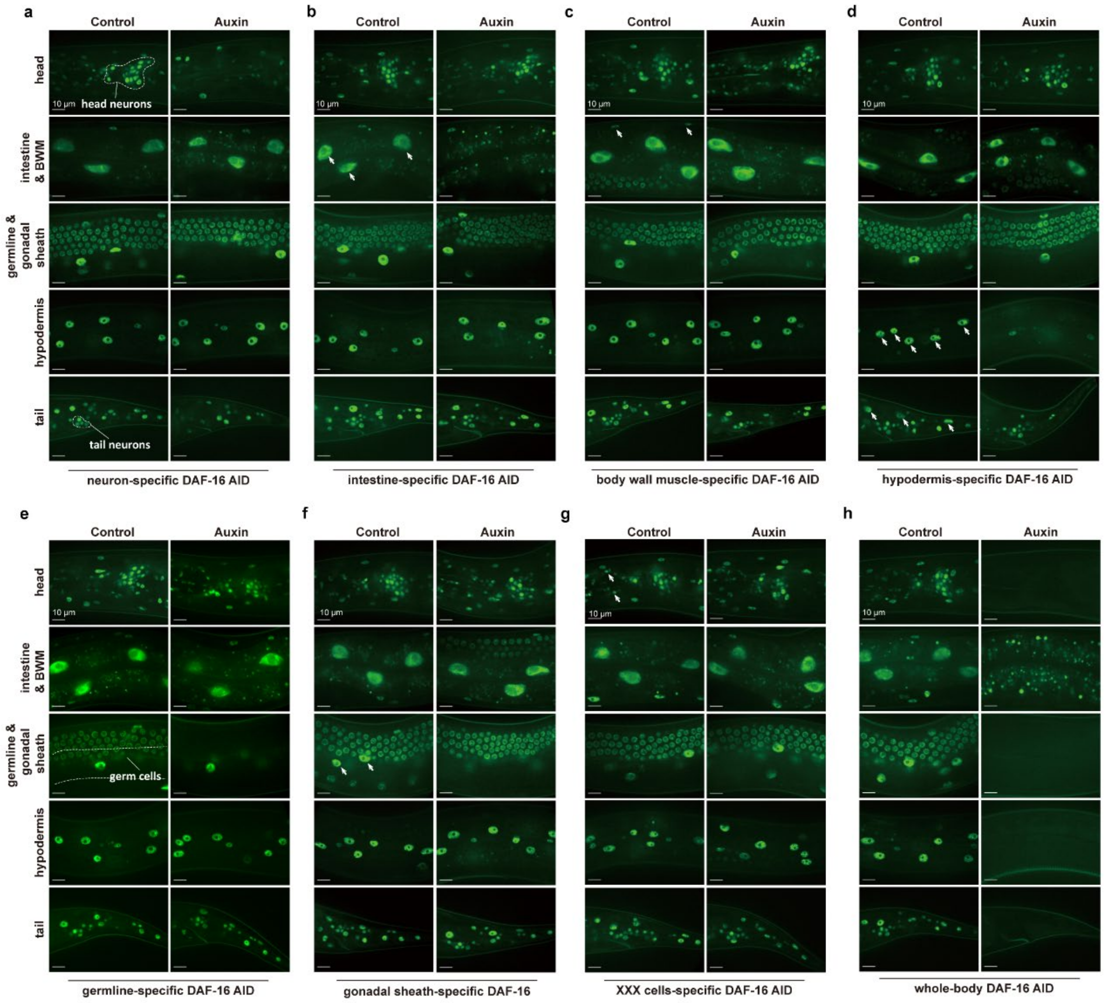

Supplementary Fig. 6. Tissue-specific AID of DAF-16::GFP::degron in *daf-2(e1370)* worms. Related to Fig. 4.

a-h Specifically degrading DAF-16::GFP::degron in the neurons (**a**), intestine (**b**), body wall muscles (**c**), hypodermis (**d**), germline (**e**), gonadal sheath (**f**), XXX cells (**g**), or whole body (**h**) with 1 mM auxin treatment for 24 hours starting from late L4 stage at 20 °C. A similar pattern of expression was observed in two independent experiments.

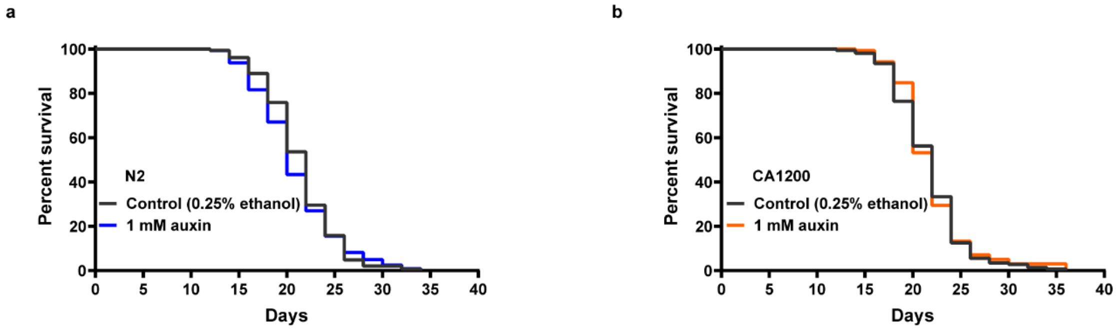

Supplementary Fig. 7. WT lifespan is not affected by 1 mM auxin treatment. Related to Fig. 3 and 4.

a-b The N2 strain (**a**) and the CA1200 strain (**b**), which expresses TIR-1::mRuby under the *eft-3* promoter were tested. *p*-values are calculated by log-rank tests. See survival statistics in Supplementary Data 1. Source data are provided as a Source Data file.

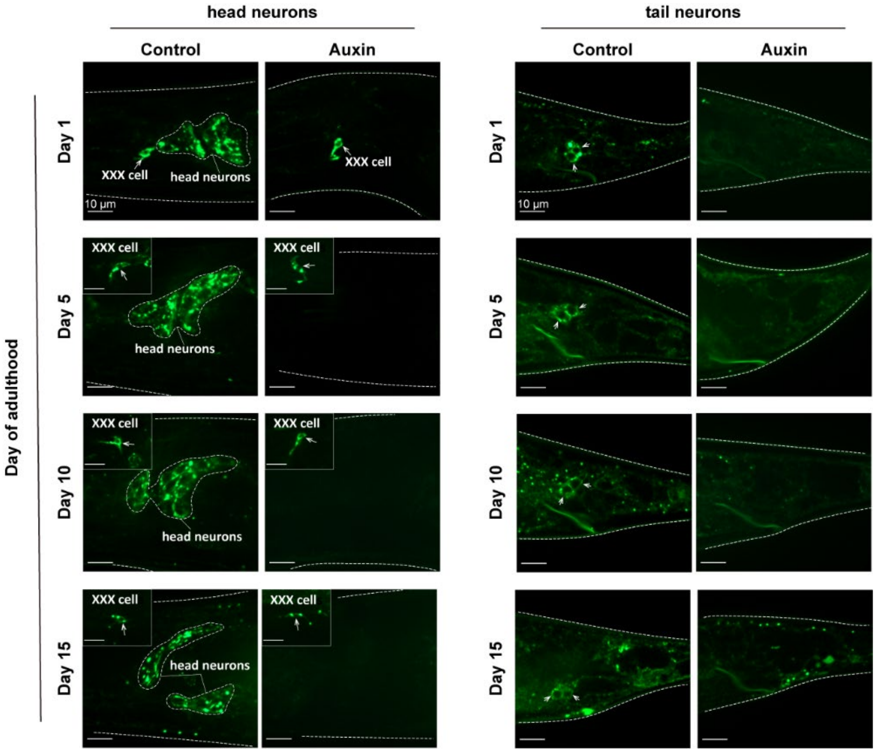

Supplementary Fig. 8. Degradation of neuronal DAF-2 by AID is sustained in old worms. Related to Fig. 3.

Neuronal DAF-2 is specifically and efficiently degraded by AID system during aging process at 20 °C. 1 mM auxin treatment was starting from late L4 stage, and images were taken at the indicated days. A similar pattern of expression was observed in two independent experiments.

a b

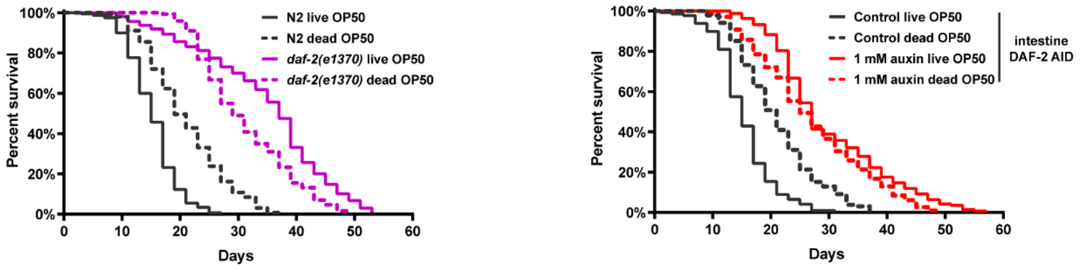

c

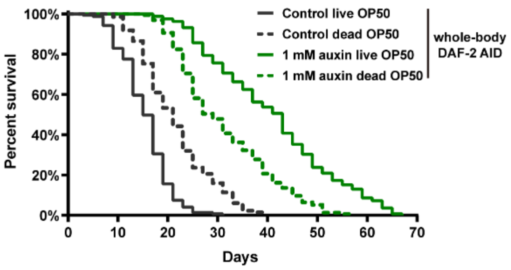

d

Variables	Hazard ratio	Reduction of hazard	*p*-value	95% confidence interval
daf-2(e1370)	0.155	0.845	<0.001	0.124-0.193
Gentamicin	0.785	0.215	0.009	0.655-0.942
Intestine DAF-2 AID	0.292	0.708	<0.001	0.240-0.356
Gentamicin	0.761	0.239	0.003	0.636-0.911
Whole-body DAF-2 AID	0.167	0.833	<0.001	0.135-0.208
Gentamicin	0.853	0.147	0.09	0.709-1.025

Supplementary Fig. 9. Survival curves of *daf-2(e1370)* and DAF-2 AID worms fed on either live or dead bacteria. Related to Fig. 3.

a-c Lifespan of *daf-2(e1370)* (**a**), intestine DAF-2 AID worms (**b**), and whole-body DAF-2 AID worms (**c**) on gentamicin-killed OP50 *E. coli*. See survival statistics of log-rank test in Supplementary Data 1. **(d)** Statistical analysis using Cox proportional hazard regression. Source data are provided as a Source Data file.

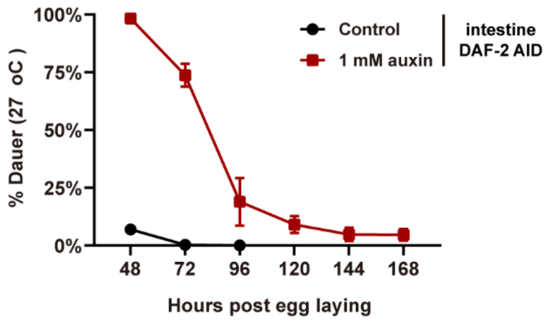

Supplementary Fig. 10. Transient dauers induced by degrading intestinal DAF-2 at 27 °C. Related to Fig. 5a.

Upon degrading intestinal DAF-2 at 27 °C, almost 99 % of the worm form dauers, but they gradually resume reproductive development within 72 hours. Data are represented as mean ± SEM of three biological replicates. Source data are provided as a Source Data file.

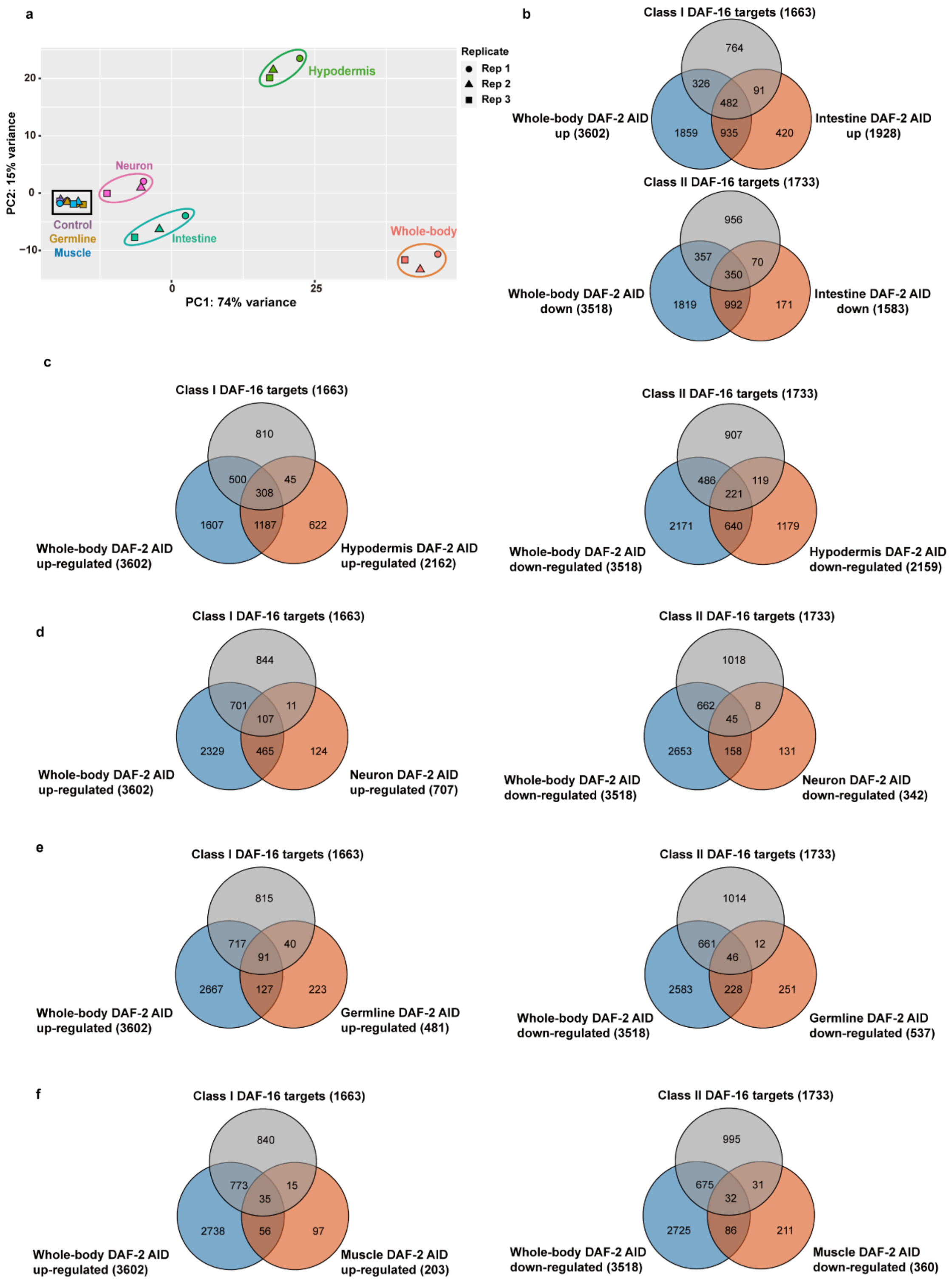

Supplementary Fig. 11. Transcriptome analysis of tissue-specific DAF-2 AID worms. Related to Fig. 6.

a Principal component analysis (PCA) shows that samples cluster with their respective genotypes. R1, R2, and R3 represent three biological replicates. **b-f** Overlap of DEGs in the whole-body DAF-2 AID worms and that in the intestinal DAF-2 AID worms (**b**), hypodermal DAF-2 AID worms (**c**), neuronal DAF-2 AID worms (**d**), germline DAF-2 AID worms (**e**), or body wall muscle DAF-2 AID worms (**f**). DEGs were defined as

adjusted *p*-value<0.001. See statistics in Supplementary Data 3. Source data are provided as a Source Data file.

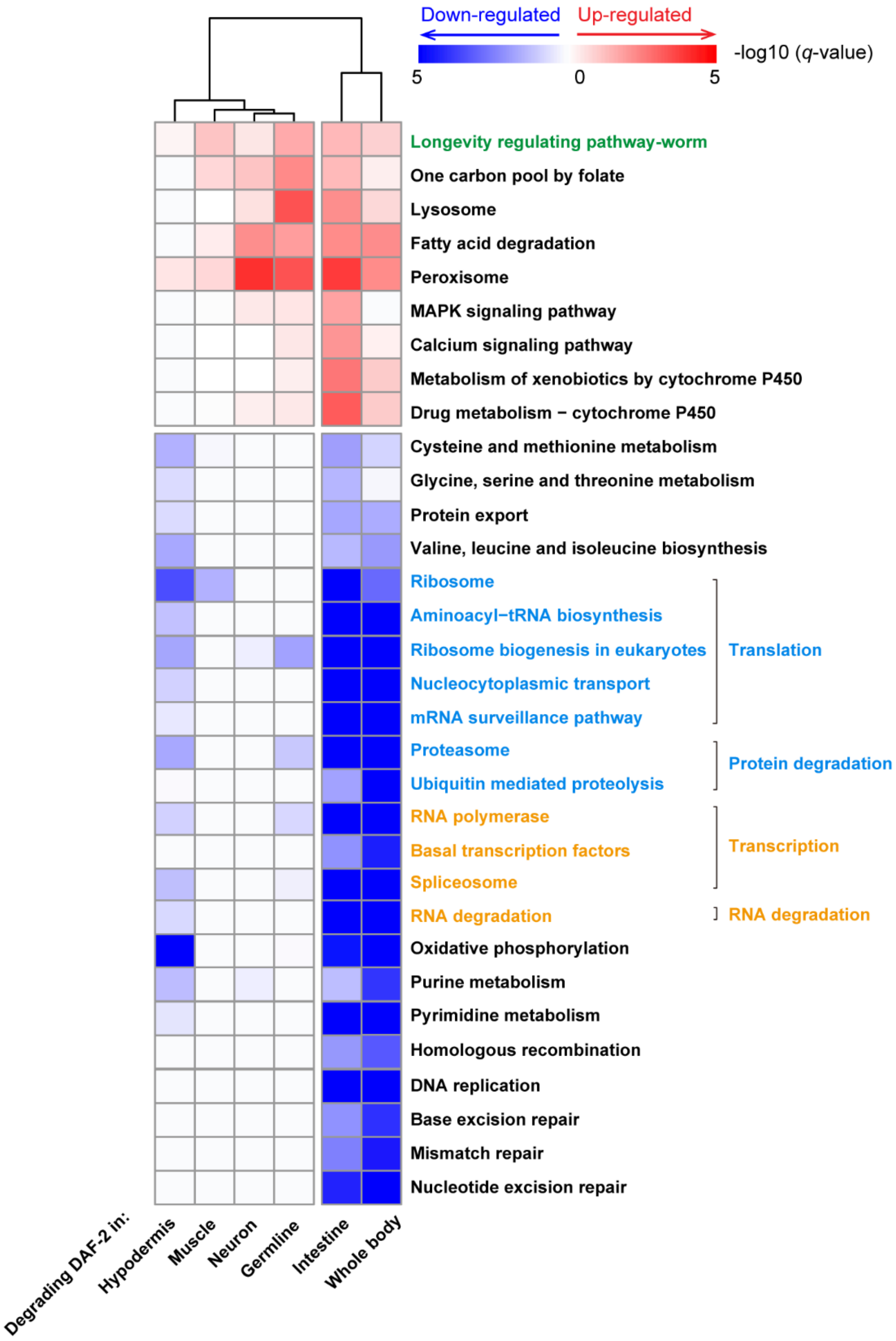

Supplementary Fig. 12. Enrichment of KEGG terms in the transcriptomic changes induced by tissue-specific DAF-2 AID, using GSEA. Related to Fig. 6. The *q*-value cutoff is 0.05 here ($q \leq 0.01$ for Fig. 6b). For multiple comparisons, adjustments were made with Benjamini–Hochberg (BH) method. Source data are provided as a Source Data file.

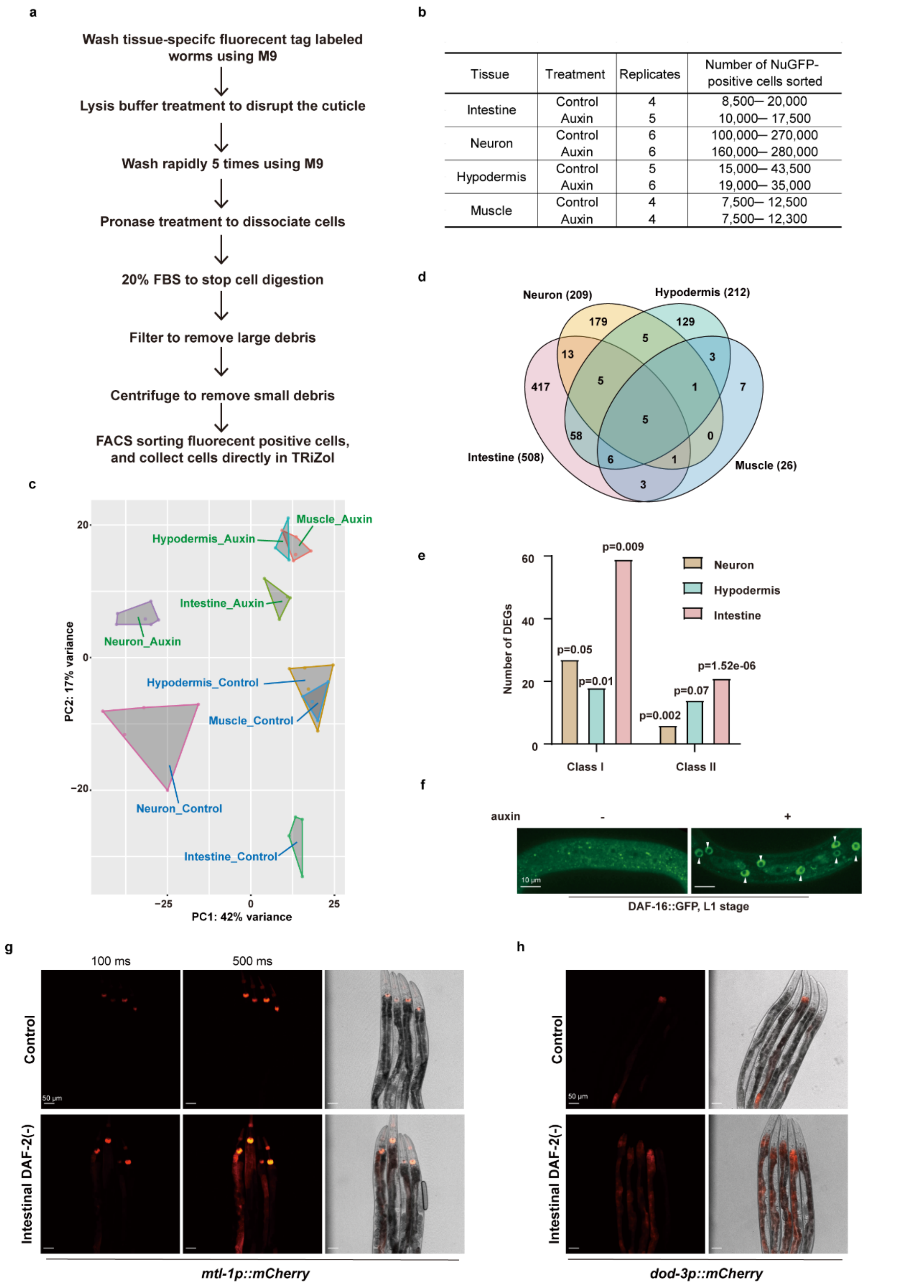

Tissue	Treatment	Replicates	Number of NuGFP-positive cells sorted
Intestine	Control	4	8,500– 20,000
	Auxin	5	10,000– 17,500
Neuron	Control	6	100,000– 270,000
	Auxin	6	160,000– 280,000
Hypodermis	Control	5	15,000– 43,500
	Auxin	6	19,000– 35,000
Muscle	Control	4	7,500– 12,500
	Auxin	4	7,500– 12,300

Supplementary Fig.13. Tissue-specific transcriptome analysis of the intestinal DAF-2 AID worms. Related to Fig. 7.

a Workflow of tissue-specific cell isolation by FACS. **b** Sample information of isolated cells for tissue-specific RNA-seq. **c** PCA analysis shows that samples cluster with their

respective tissue types as well as corresponding treatment (ethanol versus auxin). **d** Numbers of DEGs (FDR < 0.05) identified in each isolated tissue in the intestinal DAF-2 AID worms. See statistics in Supplementary Data 4. **e** Moderate enrichments of Class I and Class II DAF-16 targets are found among the hypodermal and neuronal DEGs upon degrading DAF-2 in the intestine. *p*-values are calculated using a hypergeometric test. **f** Degrading DAF-2 from the intestine induces DAF-16 nuclear accumulation in the hypodermis at the L1 larval stage. A similar pattern of expression was observed in two independent experiments. **g-h** Representative images showing that intestinal DAF-2 degradation regulates the expression of classical Class I DAF-16 targets *mtl-1p::mCherry* (**g**) and *dod-3p::mCherry* (**h**) in the same tissue. A similar pattern of expression was observed in three independent experiments. Source data are provided as a Source Data file.

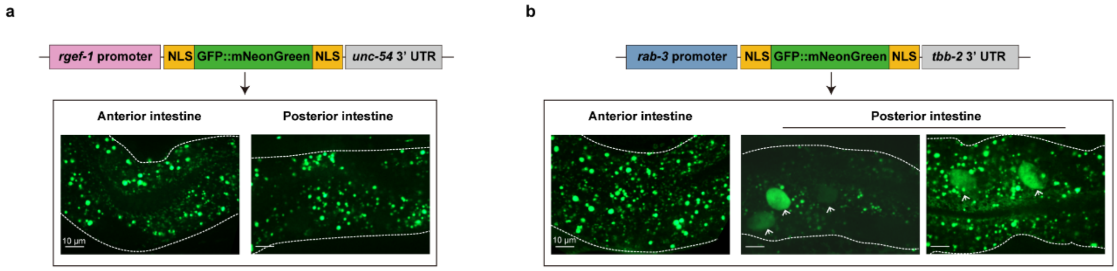

Supplementary Fig. 14. Examination of the tissue specificity of two neuron-specific promoters *rgef-1* and *rab-3*, using transgenic NuGFP reporters. Related to Fig. 3.

a *rgef-1p::NuGFP::unc-54 3'UTR* shows no leaky expression in the intestine. A similar pattern of expression was observed in two independent experiments. **b** *rab-3p::NuGFP::tbb-2 3'UTR* shows leaky expression in some posterior intestinal cells. Images were taken by dissecting intestine at adult day 1. A similar pattern of expression was observed in two independent experiments.
