# Supplementary material for: Intestine-specific removal of DAF-2 nearly doubles lifespan in Caenorhabditis elegans with little fitness cost
Source: Nat Commun. 2022 Oct 25;13:6339. doi: 10.1038/s41467-022-33850-4 (PMC9596710; doi:10.1038/s41467-022-33850-4)
Supplement: Supplementary file 3 — Description of Additional Supplementary Files [file 41467_2022_33850_MOESM3_ESM.pdf]

File Name: **Supplementary Data 1**
Description: Statistical analyses of lifespan experiments.

File Name: **Supplementary Data 2**
Description: Differentially expressed genes identified in *daf-2(e1370)* worms.

File Name: **Supplementary Data 3**
Description: Differentially expressed genes identified in each tissue-specific DAF-2 AID worms.

File Name: **Supplementary Data 4**
Description: Differentially expressed genes identified in each isolated tissue upon loss of intestinal DAF-2. Genes with FDR<0.05 are listed.

File Name: **Supplementary Data 5**
Description: Motif analysis of the differentially expressed genes of tissue-specific RNA-seq data. Significant footprintDB binding factors (e-value<0.001) are listed.

File Name: **Supplementary Data 6**
Description: Strains used in this study.

File Name: **Supplementary Data 7**
Description: Oligonucleotide sequences used in this study.
