# Supplementary material for: Intestine-specific removal of DAF-2 nearly doubles lifespan in Caenorhabditis elegans with little fitness cost
Source: Nat Commun. 2022 Oct 25;13:6339. doi: 10.1038/s41467-022-33850-4 (PMC9596710; doi:10.1038/s41467-022-33850-4)
Supplement: Supplementary file 11 — Reporting Summary [file 41467_2022_33850_MOESM11_ESM.pdf]

nature portfolio

Corresponding author(s): Meng-Qiu Dong

Last updated by author(s): Sep 10, 2022

# Reporting Summary

Nature Portfolio wishes to improve the reproducibility of the work that we publish. This form provides structure for consistency and transparency in reporting. For further information on Nature Portfolio policies, see our Editorial Policies and the Editorial Policy Checklist.

## Statistics

For all statistical analyses, confirm that the following items are present in the figure legend, table legend, main text, or Methods section.

| n/a | Confirmed | |
|---|---|---|
| ☐ | ☒ | The exact sample size (*n*) for each experimental group/condition, given as a discrete number and unit of measurement |
| ☐ | ☒ | A statement on whether measurements were taken from distinct samples or whether the same sample was measured repeatedly |
| ☐ | ☒ | The statistical test(s) used AND whether they are one- or two-sided<br>*Only common tests should be described solely by name; describe more complex techniques in the Methods section.* |
| ☒ | ☐ | A description of all covariates tested |
| ☒ | ☐ | A description of any assumptions or corrections, such as tests of normality and adjustment for multiple comparisons |
| ☐ | ☒ | A full description of the statistical parameters including central tendency (e.g. means) or other basic estimates (e.g. regression coefficient) AND variation (e.g. standard deviation) or associated estimates of uncertainty (e.g. confidence intervals) |
| ☐ | ☒ | For null hypothesis testing, the test statistic (e.g. *F*, *t*, *r*) with confidence intervals, effect sizes, degrees of freedom and *P* value noted<br>*Give P values as exact values whenever suitable.* |
| ☒ | ☐ | For Bayesian analysis, information on the choice of priors and Markov chain Monte Carlo settings |
| ☒ | ☐ | For hierarchical and complex designs, identification of the appropriate level for tests and full reporting of outcomes |
| ☒ | ☐ | Estimates of effect sizes (e.g. Cohen's *d*, Pearson's *r*), indicating how they were calculated |

*Our web collection on statistics for biologists contains articles on many of the points above.*

## Software and code

Policy information about availability of computer code

| | |
|---|---|
| Data collection | 1) The image of Figure 2d (left panel) was captured by ZEISS LSM 880 using ZEN 2 software (Carl Zeiss Inc.) , and all other confocal images were taken by the spinning-disk microscope (UltraVIEW VOX; PerkinElmer) using Volocity 6.4 software (PerkinElmer).<br>2) Applied Biosystems 7500 software (version 2.0.1) was used for collecting RT-PCR data.<br>3) The whole worm RNA-seq data was generated using an Illumina HiSeq X Ten System(BGI-Shenzhen, China).<br>4) The tissue-specific RNA-seq data was generated on BGISEQ500 platform (BGI-Shenzhen, China). |
| Data analysis | 1) RNA-seq data pre-processing: the quality of RNA-sequencing raw data was inspected using FASTQC (version 0.10.1); the reads were aligned to the C. elegans reference genome (wbcel235.97) using HISAT2 (version 2.1.0); the mapped reads that overlap with coding gene features were counted using featureCounts (version 1.6.5).<br>2) RNA-seq data analysis: RNA-seq raw counts were analyzed in RStudio (version 1.1.456) with version 3.6.1 of R. The R package DESeq2 (version 1.24.0) and edgeR (version 3.26.8) were used for differential expression analysis. The R package gage (version 2.34.0) and clusterProfiler (version 3.12.0) were used for functional enrichment (KEGG and GO) analysis. The R package ggplot2 (version 3.3.2) was used for data visualization.<br>3) Transcription factor binding motif analysis: 1.0 kb promoter regions of the DE genes were retrieved from WormBase using the Parasite Biomart tool (https://parasite.wormbase.org/biomart/martview/). The motif matrices were identified using RSAtools (http://rsat.sb-roscoff.fr/oligo-analysis_form.cgi/), and then analyzed using footprintDB (http://floresta.eead.csic.es/footprintdb/).<br>4) Statistical analyses of lifespan assays were performed using the IBM SPSS Statistics 20 software. |

For manuscripts utilizing custom algorithms or software that are central to the research but not yet described in published literature, software must be made available to editors and reviewers. We strongly encourage code deposition in a community repository (e.g. GitHub). See the Nature Portfolio guidelines for submitting code & software for further information.

## Data

Policy information about availability of data

All manuscripts must include a data availability statement. This statement should provide the following information, where applicable:

- Accession codes, unique identifiers, or web links for publicly available datasets
- A description of any restrictions on data availability
- For clinical datasets or third party data, please ensure that the statement adheres to our policy

1) All the RNA-seq raw data generated in this study have been deposited to NCBI under BioProject ID PRJNA770129.
2) The source data of figures are provided in the Supplementary Datasets and Source Data file.

# Field-specific reporting

Please select the one below that is the best fit for your research. If you are not sure, read the appropriate sections before making your selection.

[x] Life sciences [ ] Behavioural & social sciences [ ] Ecological, evolutionary & environmental sciences

For a reference copy of the document with all sections, see nature.com/documents/nr-reporting-summary-flat.pdf

# Life sciences study design

All studies must disclose on these points even when the disclosure is negative.

| | |
|---|---|
| Sample size | 1) Sample size was not predetermined using any statistical method.<br>2) We followed standard protocols and procedures used in the filed to choose the sample size. Nonetheless, the sample size we used is equal or above the average considered sufficient for each specific experimental setting. |
| Data exclusions | 1) In lifespan assays, worms that had internally hatched larvae ('bagged') or ruptured vulvae ('exploded') or crawled off the agar surface or became contaminated were censored from the analysis.<br>2) In tissue-specific RNA-seq data analysis, the outlier samples were determined by the PCA biplot of principal components 1 and 2, and then were excluded in the subsequent data analysis. |
| Replication | The findings in this study were highly reproducible and all data were from at least two independent biological replicates. |
| Randomization | In each assay (imaging, lifespan assay, dauer assay and brood size assay ), worms were randomly selected for analysis from a large population including more than 1000 worms. |
| Blinding | The investigators were not blinded during data collection, because it was crucial to know which strain is used in a particular experiment in order to optimize the time of worm transfer to auxin-added NGM plates, as various strains differ in development. |

# Reporting for specific materials, systems and methods

We require information from authors about some types of materials, experimental systems and methods used in many studies. Here, indicate whether each material, system or method listed is relevant to your study. If you are not sure if a list item applies to your research, read the appropriate section before selecting a response.

## Materials & experimental systems

| n/a | Involved in the study | |
|---|---|---|
| [x] | [ ] | Antibodies |
| [x] | [ ] | Eukaryotic cell lines |
| [x] | [ ] | Palaeontology and archaeology |
| [ ] | [x] | Animals and other organisms |
| [x] | [ ] | Human research participants |
| [x] | [ ] | Clinical data |
| [x] | [ ] | Dual use research of concern |

## Methods

| n/a | Involved in the study | |
|---|---|---|
| [x] | [ ] | ChIP-seq |
| [x] | [ ] | Flow cytometry |
| [x] | [ ] | MRI-based neuroimaging |

## Animals and other organisms

Policy information about studies involving animals; ARRIVE guidelines recommended for reporting animal research

| | |
|---|---|
| Laboratory animals | 1) Organism: Caenorhabditis elegans.<br>2) Sex: hermaphrodite<br>3) Strains: wild type N2, long-lived daf-2 mutant (e1370), and a set of genetic editing strains generated in this study were listed in the |

| | |
|---|---|
| | Supplementary Dataset 6.<br>4) Stage: experiments in this study were performed using embryonic stage, L1 stage, L2 stage, L3 stage, L4 stage, adult day 1, day 5, day 10, or day 15 worms. |
| Wild animals | This study did not involve wild animals. |
| Field-collected samples | This study did not involve samples collected form the wild. |
| Ethics oversight | No ethical approval or guidance was required in this study because only the nematode (round worm) C. elegans was used as a genetic model. |

Note that full information on the approval of the study protocol must also be provided in the manuscript.
